# Determination of Surface Stresses in X20Cr13 Steel by the Use of a Modified Hardness Measurement Procedure with Vickers Indenter

**DOI:** 10.3390/ma13214844

**Published:** 2020-10-29

**Authors:** Bogusław Hościło, Krzysztof L. Molski

**Affiliations:** Faculty of Mechanical Engineering, Bialystok University of Technology, Wiejska 45C, 15-351 Bialystok, Poland; k.molski@pb.edu.pl

**Keywords:** vickers hardness, depth sensing hardness, residual stress, finite element modelling, elastic-plastic material properties

## Abstract

The paper presents a method for estimating the value of equibiaxial stress in a surface layer of a material by using a modified hardness measurement procedure with a Vickers indenter. A certain characteristic parameter was defined and related to the surface stress. A hybrid approach, based on experimental tests and accompanied by the complementary results obtained by the finite element modelling of X20Cr13 steel in elastic–plastic range, confirmed a linear relationship between the value of the characteristic parameter and the magnitude of equibiaxial stress at the surface. This linear relationship was valid in both elastic and elastic–plastic strain range beyond the yield stress of the material.

## 1. Introduction

Fatigue of structural elements is one of the most common damage phenomena caused by a cycling loading and associated with the failure of engineering components. The fatigue process is usually related to the presence of the stress raisers, which increase both the range and the mean value of the stress cycle and cause localized destruction of the material.

Residual stresses appear as a result of various mechanical and thermal processes and may significantly affect fatigue life by changing the mean stress at the weakest zone of the structure. Many forms of the surface treatment or welding techniques produce local plastic deformation, which introduce residual stress at the outer layer of the material and change its durability. Some examples dealing with the influence of residual stresses on the fatigue initiation period in welded joints were presented by Nykanen and Björk [1], Sepe et al. [2] and Tchoffo et al. [3]. Experimental tests and numerical modelling of the influence of preheating on residual stress was investigated by Ding et al. [4] for 12CrNi2 alloy steel.

Residual stresses affect many aspects of the use of structural materials. There is also a lot of ambiguity as to the impact of residual stress on fatigue life including the fatigue crack propagation rate. In reference [5] Amjad et al. pointed out that the application of compressive overload caused by cold extrusion creates relaxation of residual stress, which improved the fatigue life of the tested material. An analysis of the impact of residual stress resulting from friction welding on fatigue strength was presented in [6]. Methods for measuring of residual stress are described by Kandil et al. in the report [7].

Furthermore, Ma et al. [8], Takakuwa et al. [9] and Tosha [10] pointed to the possibility of using microhardness measurements to determine the level of residual stresses. In [9], the authors present the problem of determining the residual stress and the yield stress in a thin layer of the material after the peening process. This type of surface treatment causes significant changes to the subsurface, increasing the fatigue strength and resistance to stress corrosion cracking.

Recently, Sajjad et al. [11] have made an attempt to relate the material characteristics obtained experimentally from the cyclic indentation test to the fatigue properties of the material.

For stress analysis various experimental techniques have been developed. Thermoelastic stress analysis (TSA) and synchrotron X-ray diffraction (SXRD) techniques are usually applied.

The level of residual stress and yield stress of the thin surface layer of the material having altered properties cannot be determined on the basis of the quasi-static tensile testing, because in such a test the average properties of the entire volume of the material are taken into account.

In the year 1951 Tabor [12] and Toit-Meyer [13] established the theoretical basis of the analysis by relating the hardness of the material to the representative strain field appearing under the indenter during the process of indentation. Based on those assumptions, Takakuwa et al. [9] experimentally determined the relationship between the residual stress and the Vickers hardness. In the cited work, the relationship between the hardness and the yield strength was also confirmed experimentally.

In the publication [14], Suresh and Giannakopoulos presented a method for determining the residual stress based on the characteristic values identified from the *P–h* curve, obtained experimentally in the process of instrumented indentation. Residual stress, according to the method, can be determined by comparing the values obtained from the reference curve *P*_0_–*h*_0_, derived for the same material without residual plastic strains, to the corresponding values for the material where residual stresses are present. The use of instrumented hardness measurements and FEM modelling for determining the residual stresses was also presented by Jang [15], Pak et al. [16] Pharr et al. [17] and Sakharova et al. [18]. Numerical modelling of the Vickers indentation testing was described by Dias et al. in [19].

Heat treatment—in particular the quenching process containing continuous cooling—introduces severe stresses of the second type [20,21]. Tempering process reduces stress values and stress gradients but does not eliminate them completely. Therefore, having a convenient tool for evaluating their values would be very useful.

There are several methods, which enable the evaluation of surface stresses below the yield stress of the material. One of them was proposed by Nishikawa et al. [22], where the authors use the classical hardness measurements with a spherical indenter and find that the maximum depth of the indentation is sensitive to the stress in the surface layer. Higher indentation depth causes larger plastic deformation under the probe, which interferes with the global strain field. In the case when the latter corresponds to the elastic–plastic range it influences the accuracy of the evaluated stress existing in the material and distorts its value. Therefore, the new method should eliminate this difficulty. The objective of the present work was to develop a method of indentation covering also the more extended stress range—beyond the yield stress of an elastic-plastic material exhibiting plastic hardening behavior in a quasi-static tensile test.

## 2. Hybrid Approach for Evaluating Surface Stress

### 2.1. Material and Method

The X20Cr13 stainless steel, also known as 1.4021 and AISI 420 steel, with the ability for thermal improvement, good ductility and machinability, is commonly used in pump components, turbine blades, shafts and surgical tools. Welding may be also performed after preheating to about 150–200 °C with recommended tempering after welding. The X20Cr13 steel is usually used after a heat treatment process, which means that residual stresses are introduced and may affect its fatigue strength and life. As mentioned earlier, many methods applied to estimate mechanical properties are based on instrumented indentation techniques. Similar tests also serve to determine residual stresses [23,24,25,26].

An approach developed in the present study is different from the one used in traditional hardness measurements where a static force of a known magnitude is applied. The difference consists of determining the stabilized force P* at which a certain constant penetration depth h* is reached during the instrumented indentation test. In order to achieve such a purpose, the Vickers pyramidal indenter has appeared to be more convenient than the spherical one. The advantages of the use of the pyramidal indenter can be specified as follows:

- versatile applications. The indentation process may be carried out to both—very hard and annealed steels, where the hardness of the X20Cr13 steel subjected to heat treatment, given as an example, covers an approximate range of 20 HRC to about 46 HRC;
- favorable ratio of depth to indentation area, adequate to reflect the average hardness of the structural components of the material;
- very clear indentation boundaries.

After performing some preliminary tests on the X20Cr13 steel subjected to heat treatment, a constant penetration depth h* = 4 μm was chosen. For such a characteristic penetration value—h*–the instrumented indentation measurements can be carried out over a wide range of material hardness, giving the reasonable range of forces P* corresponding to the measuring capacity of a micro-hardness tester.

### 2.2. Specimen, Stress Inducing Device and Experimental Results

**Figure 1:**
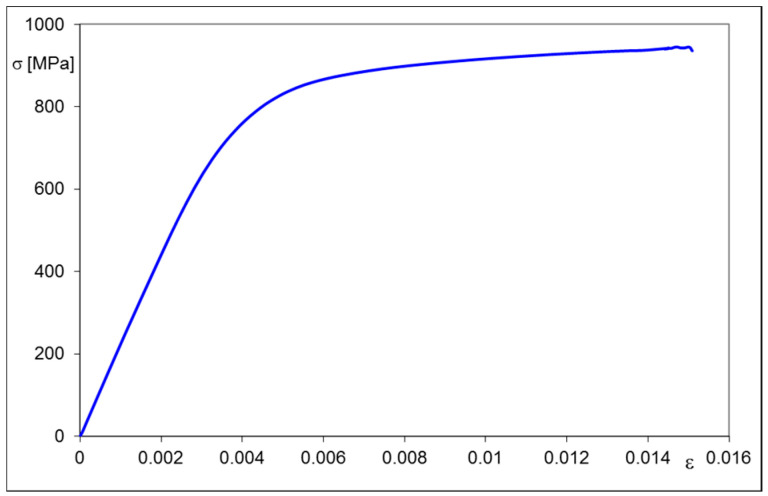
Stress–strain relationship of annealed X20Cr13 steel subjected to tensile test.

A thin circular plate of 16 mm in diameter and 0.75 mm thick was made of X20Cr13 steel. The material was annealed at 800 °C and subjected to air-cooling. A typical cylindrical specimen made of the same material and subjected to identical heat treatment as the circular one was also prepared and served for performing quasi-static tensile test. The first part of the stress–strain characteristic of the material obtained from the tensile test is depicted in Figure 1.

As a result of the test two specific values: the limit of proportionality equal 542 MPa and the proof stress equal 874 MPa were also determined.

**Figure 2:**
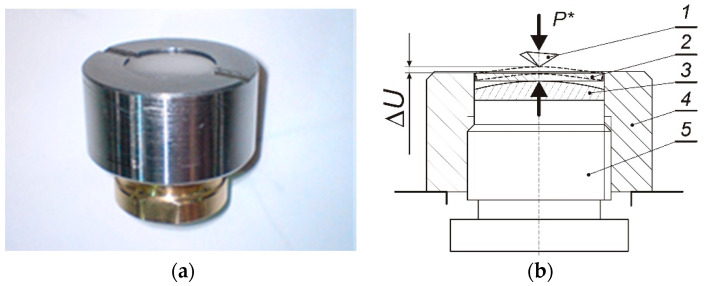
Photograph (**a**) and schematic representation (**b**) of the apparatus for inducing equibiaxial stress at the center of the circular sample subjected to bending, where: 1—tip of indenter, 2—circular sample, 3—spherical element made of bearing steel, 4—screw cap, 5—screw-in pusher, ΔU—plate deflection at the central point.

Equibiaxial stress was produced at the center of the circular plate by using a specially designed and fabricated apparatus, shown in Figure 2.

The inner circumferential fang of the screw cap supports the upper edge of the circular sample. The spherical element with relatively large radius, R = 32 mm, is loaded by the screw-in pusher and exerts a smooth contact pressure in the upward direction causing axisymmetrical bending of the sample. In this manner, an equibiaxial stress is produced at the central part of the plate and the indentation process can be easily performed in the middle of the upper surface.

The loading history consisted of four quasi-static semi-cycles and accompanying indentation tests were performed for each loaded and unloaded state of the material. Firstly, the plate was simply supported at the center by the spherical element and several indentation tests were carried out in order to obtain average *P*_0_* value as well as its standard deviation. The average magnitude of the force *P*_0_* served as a reference value for the forces *P*_i_*, identified experimentally for the next semi–cycles.

In the first loading cycle a certain pressure was exerted in the upward direction causing slight bending of the sample. Relative deflection ΔU_1_ of the outer edge of the plate was measured with respect to its center, using the Micro Combi Tester from CSM Instruments, and the value of 81.8 μm was obtained. Using the same tester, the indentation was then repeated several times to find statistical parameters of the stabilized force *P*_1_*.

Next, the plate was completely unloaded, and the permanent deflection was measured, giving the residual value of ΔU_2_ = 22.3 μm. For such a plate, simply supported by the spherical element without applying external pressure, the indentation tests were repeated and the stabilized force *P*_2_* was determined. Permanent deflection of the sample indicated that local plasticity occurred during the loading semi-cycle and that negative residual stress may be expected at the center of the upper face of the plate when the load was released.

**Figure 3:**
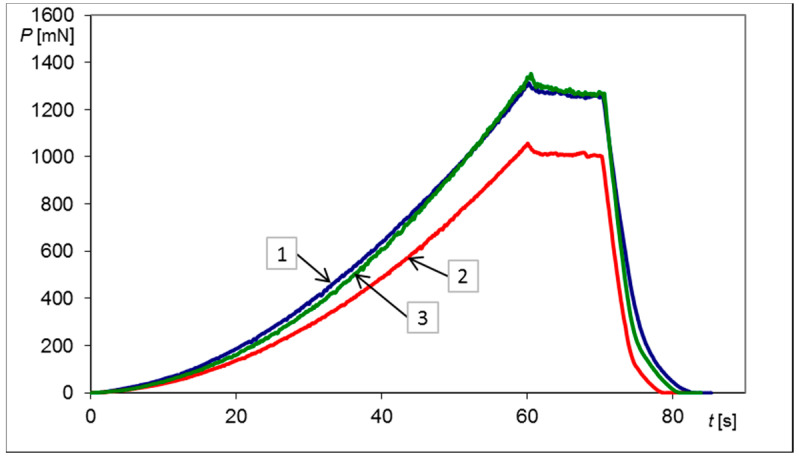
Three indentation test histories *P*–*t* performed to reach a penetration depth h* = 4 μm with a Vickers probe for a circular sample made of X20Cr13 steel, where: (1) simply supported plate, no bending—blue line, (2) after first loading—red line (plate deflection ΔU_1_ = 81.8 μm), (3) after unloading—green line (permanent deflection ΔU_2_ = 22.3 μm).

Graphical representations of the *P*–*t* histories, obtained experimentally during indentation tests for non-loaded, loaded and unloaded plate, are depicted in Figure 3.

In the following step, the second loading cycle was applied, and again the indentation tests and deflection measurements were carried out. In this case the highest deflection ΔU_3_ = 334.2 μm was obtained.

The last step consisted of releasing external load and measuring the residual deflection ΔU_4_ and the stabilized indentation force *P*_4_*. The residual deflection ΔU_4_ reached the value of 136.4 μm. In all loading and unloading semi-cycles the maximum indentation depth h* was the same and equal to 4 μm, as was previously mentioned.

**Figure 4:**
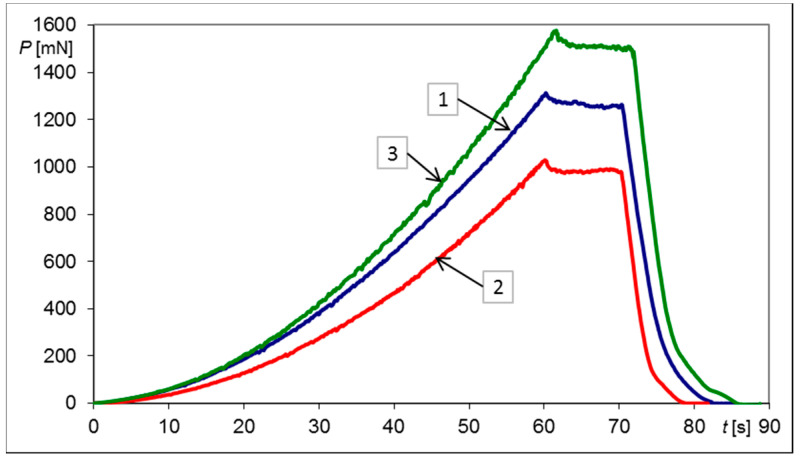
Indentation test histories *P*–*t* performed to reach a penetration depth h* = 4 μm with a Vickers probe for a circular sample made of X20Cr13 steel, where: (1) simply supported plate, no bending (reference)—blue line, (2) after second loading—red line (plate deflection ΔU_3_ = 334.2 μm), (3) after second unloading—green line (permanent deflection ΔU_4_ = 136.4 μm).

Graphical representation of the indentation test histories for the second loading and unloading semi-cycles together with the reference indentation *P*-*t* history is shown in Figure 4.

The results of all these measured quantities, complemented with additional statistical parameters, instrumented hardness values and the differences between the stabilized forces Δ*P*_i_* = *P*_i_* − *P*_0_ corresponding to particular semi-cycles, are summarized in Table 1.

### 2.3. Numerical Modelling Using the Finite Element Method

**Figure 5:**
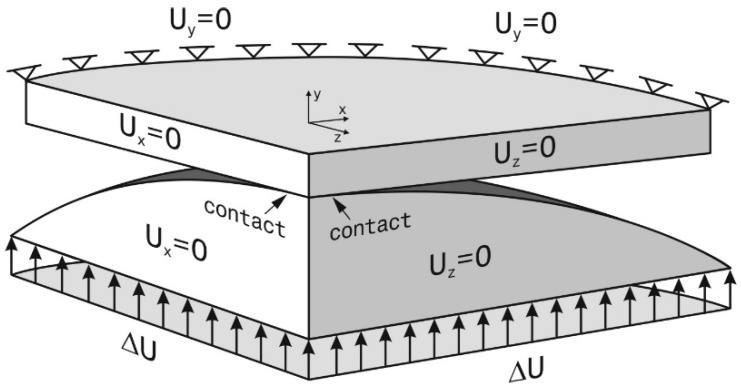
Displacement boundary conditions of the FEM model.

Numerical analysis based on the Finite Element Method was carried out in order to determine the relationship between the deflection of the plate, bent by the spherical pusher, and the equibiaxial stress at the center of the upper surface of the sample. The ANSYS R16.2 Multiphysics program and Solid185 tetrahedral finite element containing eight nodes were used. Due to the axial symmetry of the problem only one quarter of the body was modelled. Symmetrical, normal displacement boundary conditions equal zero were applied to both lateral, perpendicular sides of the plate and the pusher (Figure 5).

**Figure 6:**
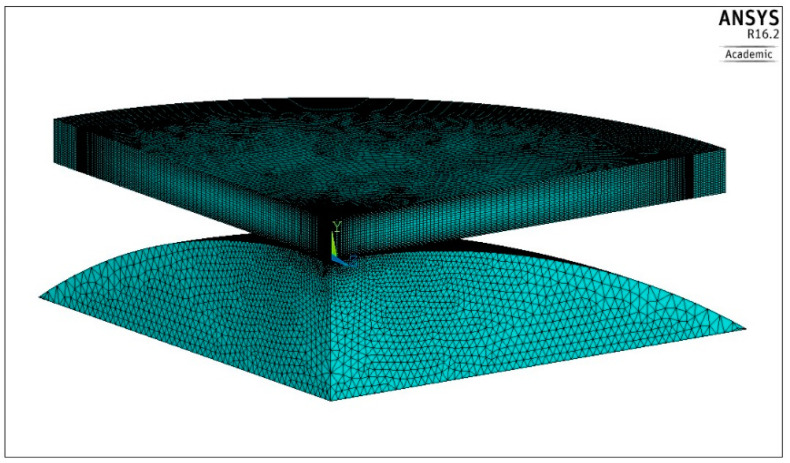
Shape of the modeled body and the finite element mesh.

Approximately 708,000 finite elements were used for modelling the plate and about 341,000 finite elements covered the volume of the spherical pusher. The modelled shape of the body with the finite element mesh is shown in Figure 6.

The characteristic of the elastic-plastic material, shown in Figure 1, was incorporated in the program as a multilinear kinematic model. Finally, the option of small deformations was chosen. The problem was considered as a non-linear, frictionless contact case with the possibility of detachment; therefore, two additional types of complementary finite elements—Targe170 and Conta174—were used over the expected contact zone.

**Figure 7:**
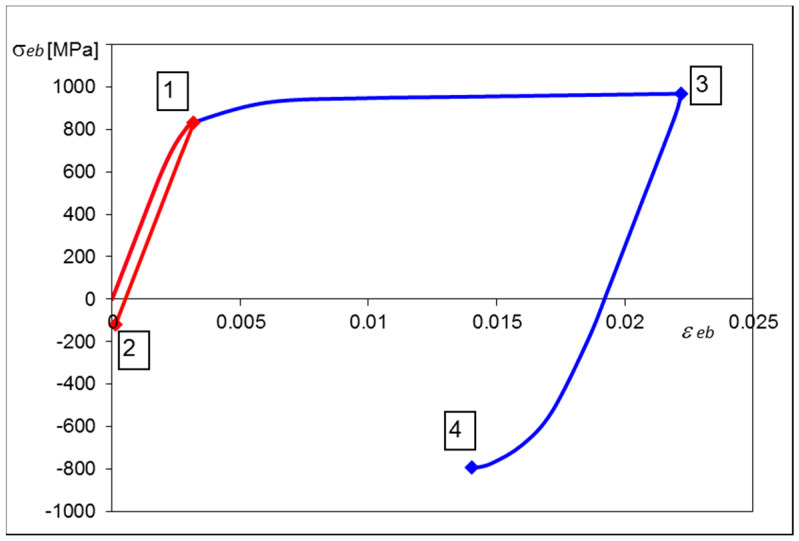
Equibiaxial stress–strain history at the surface center. Numerical FEM solution.

The loading conditions were represented by the sequence of the four displacements applied to the spherical pusher and corresponding to the deflections ΔU_i_ obtained experimentally in the previous tests. Each prescribed displacement value was reached using the iterative procedure and for each step the calculated data were recorded. In this manner the biaxial stress–strain history at the central point of the upper plate surface was found and shown in Figure 7, where particular numbers correspond to the subsequent semi-cycles.

Particular points, number 1 and 3, shown in Figure 7, represent stress and strain values produced in the middle of the plate surface as a result of bending caused by the spherical pusher. Both remaining points, number 2 and 4, represent residual stress and strain values after releasing the external load.

**Figure 8:**
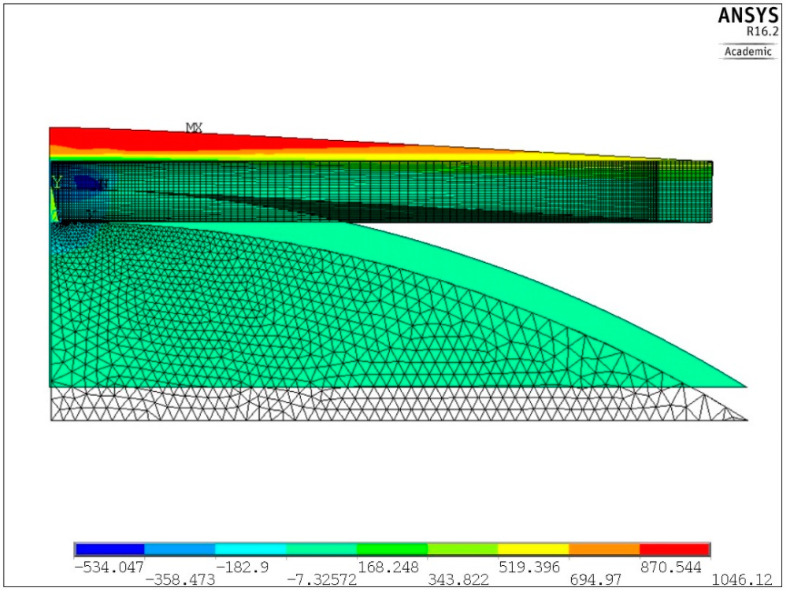
Distribution of the first principal stress *S*_1_ produced by the second loading semi-cycle, for ΔU_3_ = 334.2 μm.

**Figure 9:**
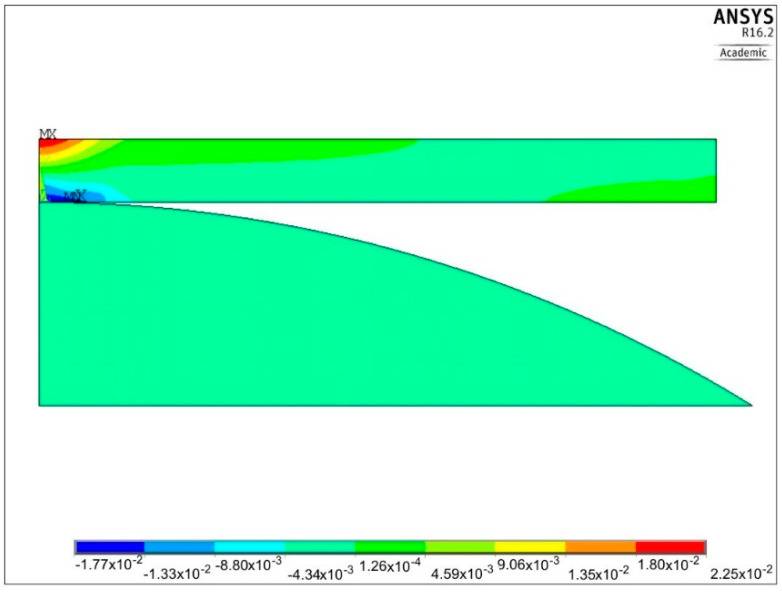
Distribution of the first principal strain ε_1_ produced by the second loading semi-cycle, for ΔU_3_ = 334.2 μm.

One example of the numerical solution of the loaded plate is shown in Figure 8 and Figure 9. Distribution of the first principal stress *S*_1_ over the cross section of the plate, corresponding to the second loading semi-cycle, is shown in Figure 8, for the deflection ΔU_3_ equal 334.2 μm. Equibiaxial stress produced at the center of the upper surface, identified from the central finite element node, was equal to 969 MPa (point 3 in Figure 7).

Distribution of the first principal strain ε_1_ for the same loading case is depicted in Figure 9. The maximum strain value, corresponding to the equibiaxial stress *S*_1_ = 969 MPa at the center of the plate, equals 0.0225 (point 3 in Figure 7).

**Figure 10:**
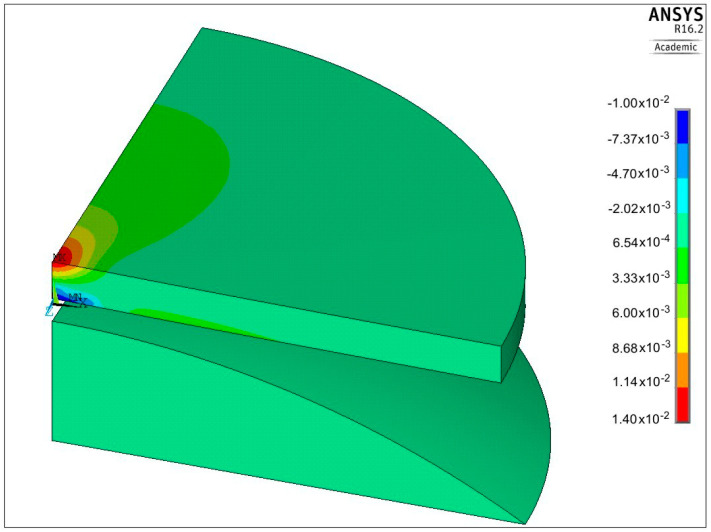
Permanent strains ε_x_, interpreted as residual ones, obtained after unloading, corresponding to the point number 4 shown in Figure 7.

Another example of the residual strains ε_x_, corresponding to the semi-cycle number 4, is presented in Figure 10. The maximum value of such a strain lies at the center of the plate and equals 0.014.

Numerical values of stresses σ_eb_ and the characteristic Δ*P*_i_* parameters, obtained from the FEM solutions and indentation tests, are given in Table 2, together with the appropriate semi-cycle numbers.

**Figure 11:**
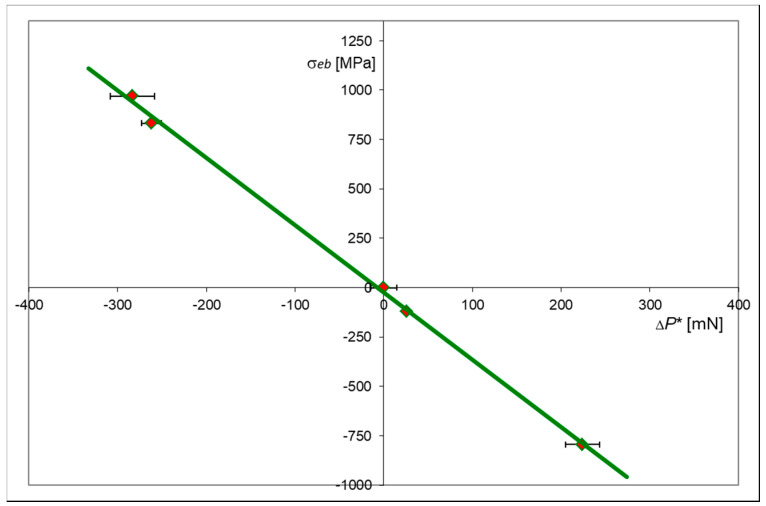
Relationship between equibiaxial stress σ_eb_ and the change in indentation force ΔP* at a constant penetration depth h* = 4 μm. Annealed X20Cr13 steel.

These numerical data were used to determine the relationship between the values of equibiaxial stress σ_eb_ and the changes in the force, Δ*P*_i_*, required to penetrate the indenter at a predetermined depth h*. Graphical representation of such a relationship is shown in Figure 11.

It is clearly seen that the relationship between the stresses σ_eb_ and the changes in indentation force ΔP* is linear. This relation is valid in both elastic and elastic–plastic range for relatively high values of the plastic strain.

## 3. Conclusions

The indentation method presented above, based on the Vickers probe together with the instrumented indentation procedure for a constant indentation depth h* = 4 μm, has appeared to be very effective in determining the magnitude of the equibiaxial stresses at the surface layer of the X20Cr13 material and may be especially useful in identifying residual stresses. Constant indentation depth h* makes it possible to measure material resistance, represented physically by external force P*, and maintaining similar penetration conditions while applying the probe to a given depth. Such a measure is more convenient, because the respective stabilized force P* can be obtained directly from the *P*-*t* graph, instead of searching for dimensions of the indented area.

Furthermore, the range of values of the characteristic force P* is convenient. As it can be seen in Table 1 and in Figure 3 and Figure 4, the magnitudes of forces applied on the indenter varied between 1 N and 1.5 N, corresponding well to the measuring capacity of a micro-hardness tester. Even for harder materials than annealed X20Cr13 steel, the Vickers indenter and the micro-hardness tester can be easily used. Therefore, the assumed constant penetration depth h* = 4 μm seems to be the right compromise from the point of view of the force applied and the indent size.

A hybrid approach based on experimental studies and accompanied by the complementary results obtained by the finite element modelling of the X20Cr13 steel in the elastic–plastic range, confirmed a linear relationship between the value of the characteristic parameter ΔP* and the magnitude of equibiaxial surface stress σ_eb_. This linear relationship is valid in the elastic and elastic–plastic strain range, beyond the yield stress of the material.

Once the material characteristic, shown in Figure 11, is obtained, it can serve as a pattern to determine equibiaxial surface stresses in any element made of the same material.

## Figures and Tables

**Table 1:** Experimental results obtained using the Micro Combi Tester and complemented by statistical parameters. Material: annealed X20Cr13 steel. Constant penetration depth h* = 4 μm.

Quantity	Units	Initial State	First Loading Semi-Cycle	First Unloading Semi-Cycle	Second Loading Semi-Cycle	Second Unloading Semi-Cycle
*i*-th semi-cycle	–	0	1	2	3	4
ΔU_i_	μm	0.0	81.8	22.3	334.2	136.4
*P*_i_*	mN	1259.5	997.5	1285.1	976.4	1483.6
Std. Dev. *P*_i_*	mN	22.0	15.1	19.3	11.3	25.0
Δ*P*_i_*	mN	0.0	−262.0	25.6	−283.1	224.1
H_IT_	MPa	3403.8	2559.8	3283.1	2508.2	3912.6
Std. Dev. H_IT_	MPa	137.9	115.5	44.3	82.9	213.0

**Table 2:** Equibiaxial stress and corresponding change of forces Δ*P*_i_*.

i-th Semi-Cycle	1	2	3	4
σ_eb_	833 MPa	−119 MPa	969 MPa	−793 MPa
Δ*P*_i_*	−262.0 MPa	25.6 MPa	−283.1 MPa	224.1 MPa

## Nomenclature

H_IT_	instrumented hardness
*P**	characteristic stabilized force corresponding to a specified penetration depth h*
*P*_0_*	characteristic stabilized reference force *P**
*P*_i_*	characteristic stabilized force *P** corresponding to the i-th semi-cycle
*h*	indenter penetration depth
h*	constant penetration depth (4 μm) used to determine the characteristic parameter *P**
Δ*P*_i_*	difference between stabilized forces Δ*P*_i_* = *P*_i_* − *P*_0_* corresponding to the i-th semi-cycle
ΔU	relative deflection of the plate between the center and the outer supported edge
ΔU_i_	relative deflection ΔU corresponding to the i-th semi-cycle
ε	strain
ε_eb_	equibiaxial strain
σ	stress
σ_eb_	equibiaxial stress
